# A New Intermittent Pumping Design for Fluid Shortage Wells

**DOI:** 10.1038/s41598-020-59094-0

**Published:** 2020-02-13

**Authors:** Xingyuan Liang, Fujian Zhou, Tianbo Liang

**Affiliations:** China University of Petroleum-Beijing, Beijing, China

**Keywords:** Solid Earth sciences, Energy science and technology, Engineering, Mathematics and computing

## Abstract

Low oil price requires oil companies to reduce costs and increase benefits. The wells with deficient fluid supplies approximately account for 20–30% of all producing wells, and this situation is even worse in the old oilfields. Intermittent production is an effective way to reduce the cost and increase the system efficiency to overcome the shortage of oil supply from the reservoir. The key is to optimize the intermittent pumping scheme, i.e., to design reasonable shut-in and operating periods. In this study, this is achieved using the dynamic change of the fluid level in the wellbore. From the electrical power curve to the dynamometer card, the dynamic drop of the fluid level can be obtained, and thus the optimal operation time of the well; at last, from the inflow performance of the well, the optimal shut-in period can be obtained. This method shows a good application in the field through a case study.

## Introduction

To face the severe situation with the low oil prices, most oil field operators have taken actions to reduce the expenditure and improve their benefits. There are a lot of wells experiencing a fluid shortage in China, and the intermittent pumping can be an effective solution for these wells^1^. When operating the intermittent pumping, the pump runs when liquid in the annulus is sufficient, while it stops when the liquid in the annulus is insufficient^2^. This operation can reduce the energy consumption, improve the pump efficiency, and alleviate the abrasion between the pump and casing. To ensure a successful intermittent pumping, the key is to determine the duration of operation and shut-in periods. Currently, this can be achieved using four methods as follows.

The first method is to use the fluid-buildup curve^3,4^. The optimal operation and shut-in period can be obtained by analyzing the good selected and the characteristics of the buildup curve of each selected well^5^. And it cannot be calculated automatically. The second method is to use the production decline curve^6,7^. According to the porosity of reservoir rock and the production history of the well, the intermittent-pumping can be designed based on the distribution of annulus inflow performance and the buildup test^7^. Many factors have an impact on the curve, so the accuracy is needed to be further enhanced. The third method is to use the economical limitation, where the optimal period is obtained by conducting the material balance and break-even analysis^8^. But there are many parameters involved. The last method is building the analytical model, where the intermittent pumping scheme is obtained by using the inflow performance and the material balance of the well. And this method also needs a liquid recovery curve. Most of the fluid shortage wells in the oil field are operated at the same intermittent pumping scheme for a long period time. However, inflow performance is different for each well in different periods, and thus the intermittent pumping should be different for each well and changed according to production needs^9^. Based on material balance, exponential expressions of the shut-in and production periods have been established^10^. Besides, based on the production test and the variation rate of the casing pressure, the intermittent time can be determined^11^. A reduced pressure has been defined and an initial intermittent pumping scheme has also been figured out. Based on the scheme, a model on the change of annulus fluid level with time has been built. Moreover, the fluid buildup curve and the optimized intermittent pumping for fluid shortage wells have been obtained^1^. The dynamic fluid level is observed by the acoustic waves in the above methods. However, the collecting data of acoustic waves by a human is not continuous. Furthermore, monitoring data is important, which can help us adjust the intermittent pumping scheme in real-time.

In this study, a new method of intermittent pumping has been developed. First, gathered in real-time, the electrical parameters are used to interpret the surface dynamometer card; secondly, dynamic fluid depth is solved by the downhole dynamometer card, which can be converted from the surface dynamometer card; thirdly, during the shut-in period, the buildup curve can be drawn through the mathematical relationship between the inflow performance of wells and submergence; finally, the intermittent pumping can be obtained by the slope method.

## Method: Optimization of Intermittent Pumping Scheme

### Using electrical power curve to get the surface dynamometer card

A working pumping unit (PU) can show different kinds of working patterns. Powered by electricity, the motor of the PU drives the horsehead up and down. Since the electrical energy is converted into mechanical energy, any change of stress at suspension point can be detected from the consumption of electrical energy. For example, if PU gets stuck in vertical motion or the viscosity of oil becomes more viscous, the stress at suspension point would increase, increasing of the input power. On the contrary, if PU has leakage or experiences a blowout, energy consumption would decrease. Therefore, the working conditions can be interpreted from the energy consumption curve.

By studying the relationship of power, torque, and loading, a mathematical model between loading and electrical power can be established.

1 $$E=9549\frac{{N}_{r}\eta i}{{n}_{m}}$$

2 $$E=[\frac{a}{b}{\rm{P}}-\frac{c}{b}{{\rm{W}}}_{b}(\cos \,\theta -\frac{c}{a}\frac{{a}_{A}}{g})]\frac{r\,\sin \,a}{\sin \,\beta }-{W}_{c}^{{\prime} }r\,\sin \,\varphi $$

The relationship between electrical power and loading is then calculated from the above two formulas.

3 $$P=f({{\rm{N}}}_{{\rm{r}}})$$

The displacement curve and surface dynamometer card are interpreted from the crank angle and the electrical power curve, respectively.

### Getting dynamic fluid level from surface dynamometer card

**Figure 1:**
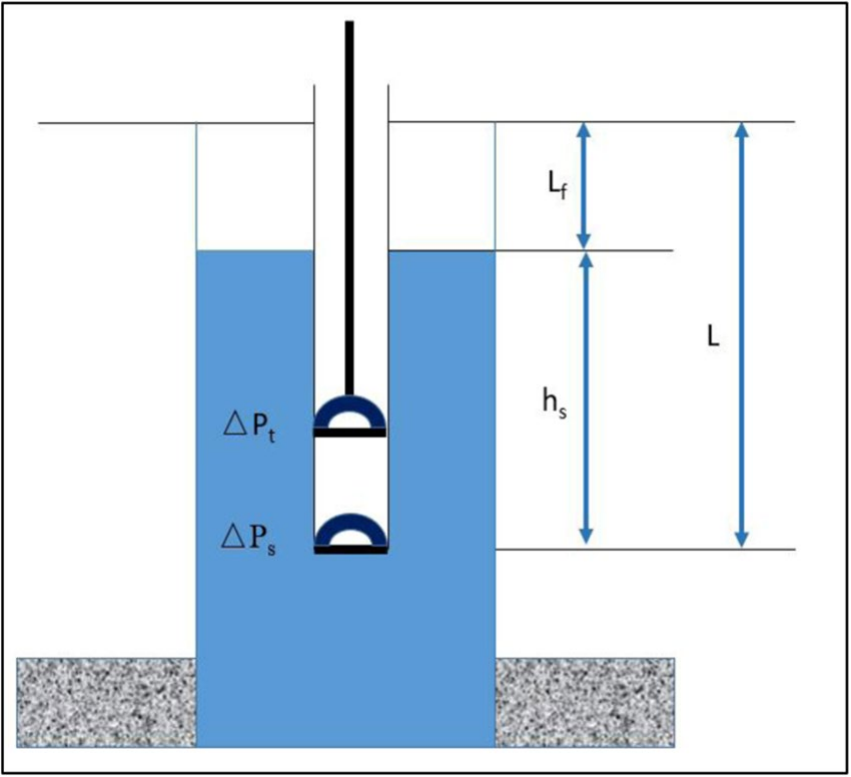
Locations of static and dynamic levels.

Using the Gibbs equation can interpret the pump dynamometer card from the surface dynamometer card^12^. The dynamic fluid level is defined as the height of fluid in the annulus (as shown in Fig. 1).

When the horsehead is at the bottom dead center, the stress at the suspension point (F_d_) can be expressed as the Eq. (4).

4 $${F}_{d}=[{{\rm{h}}}_{s}{\rho }_{l}g+({P}_{c}-{\Delta {\rm{p}}}_{s})\times {10}^{6}]{A}_{p}$$

When the horsehead is at the top dead center, the stress at the suspension point (F_u_) can be expressed as the Eq. (5).

5 $${F}_{u}=[L{\rho }_{l}g+({P}_{t}+\Delta {p}_{t})\times {10}^{6}]{A}_{p}$$

The dynamic depth can be calculated as Eq. (6).

6 $${L}_{f}=L-{h}_{s}$$

The resistance of travel (ΔP_t_) and affix valve (ΔP_s_) can be calculated as the Eq. (7).

7 $$\Delta {p}_{t}=\Delta {p}_{s}=\Delta p=\frac{{\rho }_{l}{\nu }_{f}^{2}}{2{\xi }^{2}}$$

The Eq. (8) can be solved from Eqs. (4) to (5).

8 $${L}_{f}=\frac{({F}_{u}-{F}_{d})-{10}^{6}({{\rm{P}}}_{{\rm{t}}}-{{\rm{P}}}_{{\rm{c}}}){{\rm{A}}}_{{\rm{p}}}}{{{\rm{A}}}_{{\rm{p}}}{\rho }_{l}g}-\frac{{10}^{6}{\nu }_{f}^{2}}{{\xi }^{2}g}$$

The Eq. (8) also can be expressed as the following equation.

9 $${L}_{f}=\frac{\Delta W-{10}^{6}({{\rm{P}}}_{{\rm{t}}}-{{\rm{P}}}_{{\rm{c}}}){{\rm{A}}}_{{\rm{p}}}}{{{\rm{A}}}_{{\rm{p}}}{\rho }_{l}g}-\frac{{10}^{6}{\nu }_{f}^{2}}{{\xi }^{2}g}$$

Therefore, the dynamic fluid level can be obtained from the loading and displacement of the surface dynamometer card.

### Variation law of the submergence

#### Declining law of the submergence

**Figure 2:**
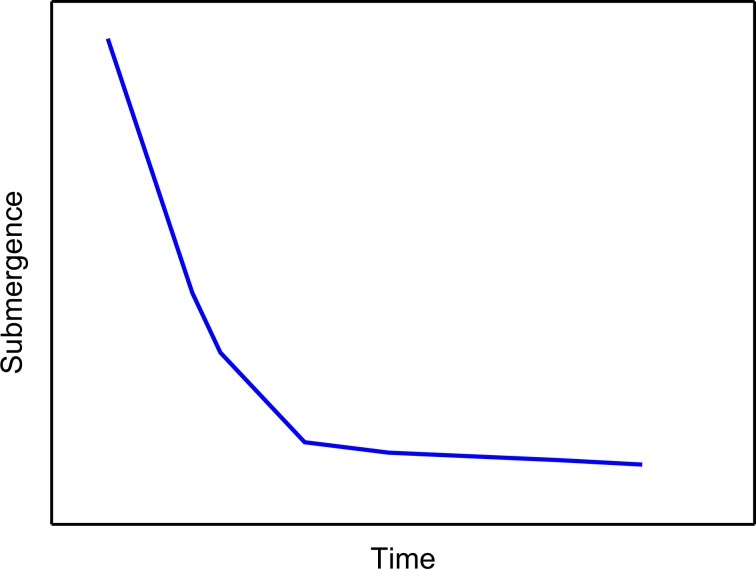
The decreasing curve of submergence during the operation period.

The submergence depth of a pump in the wellbore fluid equals the depth of the pump subtracting the dynamic fluid depth. The submergence is high when PU starts working. The submergence decreases fast at the beginning of the pumping suction. When the submergence falls to a certain low level, the fluid in the pump would also be at a low degree. The typical decreasing curve of submergence is shown in Fig. 2.

#### Increasing law of the submergence

**Figure 3:**
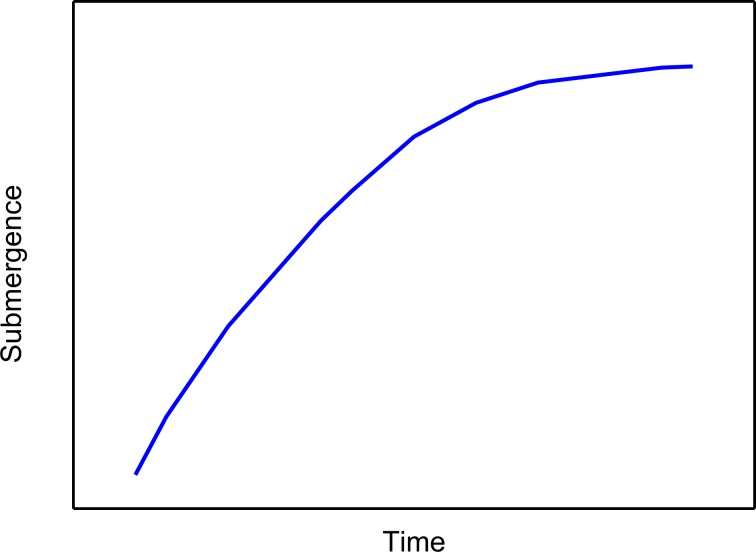
The increasing curve of submergence during the shut-in period.

During the shut-in period when PU stops working, the increasing law of submergence can be studied through the inflow performance of the well^2^. The pressure difference between surface and bottom-hole forces fluid flowing into the bottom of the well. And raising fluid in the annulus leads to an increase of submergence. The increase of dynamic fluid level further enhances the bottom-hole pressure, which reduces the pressure difference and the fluid flowing into the well. At the end of the shut-in period, the submergence approaches a plateau. It turns out that a longer shut-in period reduces the cumulative production. The submergence of fluid shortage wells firstly increases fast then tends to a stable position in the end. The typical increasing curve during the shut-in period is shown in Fig. 3.

### Determination of well-operation period

Once the decreasing curve of submergence is obtained from the monitored electrical curve, the slope of the decreasing region is calculated for determining the optimal period of good operation. The absolute value of the slope within a chosen time interval is calculated since the beginning of the well-operation. This is repeated until the absolute value of the slope is smaller than a pre-determined value (ε, as described later), and the period till this moment is recorded as the working time T_1_ for operating the well. The details are shown as follows.

**Figure 4:**
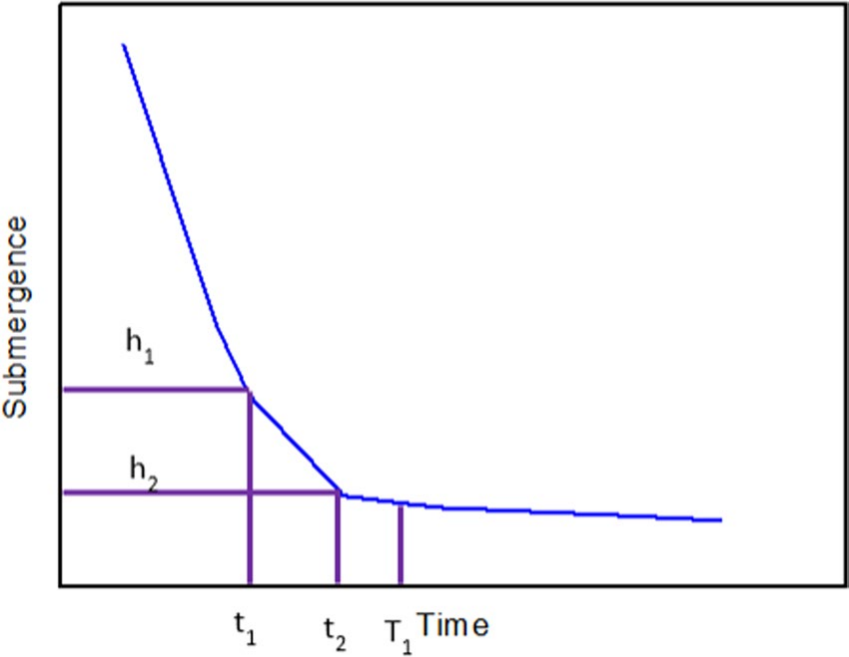
Determination of well-operation period from the decreasing curve of submergence.

After the obtained submergence decreasing curve is evenly divided into N intervals, the slope of each interval can be determined from coordinates of two adjacent points are recorded ((t_1_, h_1_) and (t_2_, h_2_) in Fig. 4). PU stops working when the absolute value of the slope is small than a certain value (ε_1_), i.e.; $|\frac{{h}_{2}-{h}_{1}}{{t}_{2}-{t}_{1}}|\le {\varepsilon }_{1}$; and this moment is recorded as T_1_ as shown in Fig. 4. For the first time, the ε_1_ can be obtained by the former experience, then the ε_1_ would be corrected by the method in this paper. Thus, the former ε_1_ would be used to help determine the next new ε_1_ in the end.

**Figure 5:**
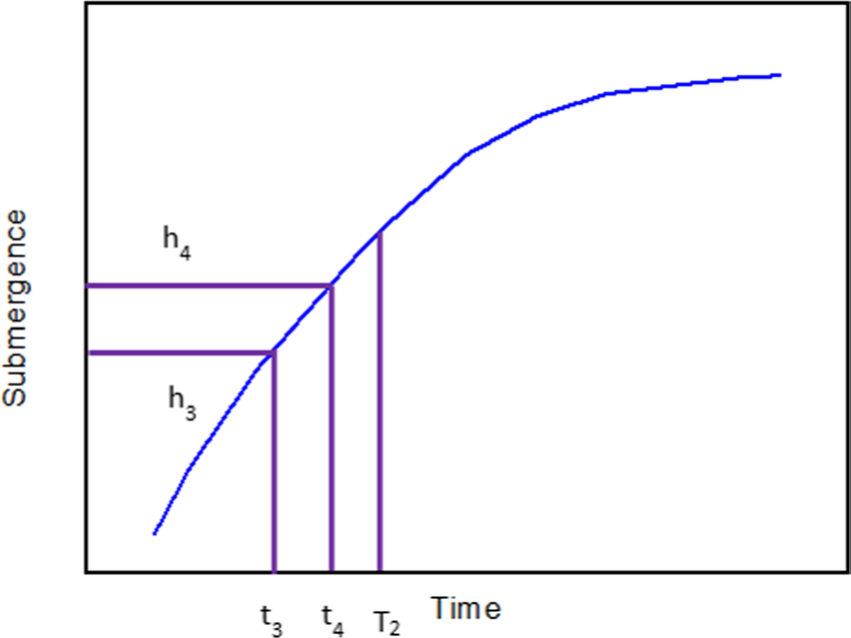
Determination of well-shut-in period from the increasing curve of submergence.

### Determination of well-shut-in period

The buildup law of fluid depth can be interpreted from the bottom hole inflow performance. Because of three-phase (oil, gas, and water) in the late production period, here the Petrobras way is introduced to calculate the production^13^.

10 $$d{p}_{wf}=\rho gdh$$

11 $$dh=\frac{Qdt}{s}$$

The Eq. (12) can be solved from the Eqs. (10) to (11).

12 $$\frac{d{p}_{wf}}{Q}=\frac{\rho g}{s}dt$$

Besides, the total liquid output is calculated as Eq. (13).

13 $$Q=(1-{{\rm{f}}}_{{\rm{w}}}){{\rm{q}}}_{{\rm{oil}}}+{{\rm{f}}}_{{\rm{w}}}{{\rm{q}}}_{{\rm{water}}}$$

The total oil output is calculated as Eq. (14).

14 $${q}_{oil}={q}_{b}+({{\rm{q}}}_{{\rm{omax}}}-{{\rm{q}}}_{{\rm{b}}})[1-0.2(\frac{{p}_{wf}}{{p}_{b}})-0.8{(\frac{{p}_{wf}}{{p}_{b}})}^{2}]$$

The total water output is calculated as Eqs. (15) and (16).

15 $${q}_{water}={J}_{1}({{\rm{p}}}_{{\rm{r}}}-{{\rm{p}}}_{{\rm{wf}}})$$

16 $${J}_{1}=\frac{{q}_{b}}{{p}_{r}-{p}_{b}}$$

Finally, Eq. (17) can be obtained from the Eqs. (12) to (16).

17 $$-Bdt=\frac{d{p}_{wf}}{W{p}_{wf}^{2}+Y{p}_{wf}+Z}$$

where

18 $$B=\frac{\rho g}{s}$$

19 $$W=\frac{0.8(1-{{\rm{f}}}_{{\rm{w}}})({{\rm{q}}}_{{\rm{omax}}}-{{\rm{q}}}_{{\rm{b}}})}{{p}_{r}^{2}}$$

20 $$Y=\frac{0.2(1-{{\rm{f}}}_{{\rm{w}}})({{\rm{q}}}_{{\rm{omax}}}-{{\rm{q}}}_{{\rm{b}}})}{{p}_{r}}+{f}_{w}\frac{{q}_{b}}{{p}_{r}-{p}_{b}}$$

21 $$Z=-[(1-{{\rm{f}}}_{{\rm{w}}}){{\rm{q}}}_{{\rm{b}}}+{{\rm{f}}}_{{\rm{w}}}\frac{{q}_{b}}{{p}_{r}-{p}_{b}}{{\rm{p}}}_{r}]$$

and then

22 $${p}_{wf}=\frac{\frac{2M}{1+{e}^{-M({\rm{Bt}}+C)}}-Y-M}{2W}$$

23 $$M=\sqrt{{Y}^{2}-4WZ}$$

The C in the equation can be solved at the condition of t = 0 and the submergence at the beginning of the shut-in period.

Therefore, the relationship between submergence and time is also expressed as follows.

24 $$H=\frac{{p}_{wf}}{\rho g}+{H}_{pump}-{H}_{0}$$

25 $$H=\frac{\frac{2M}{1+{e}^{-M({\rm{Bt}}+C)}}-Y-M}{2W\rho g}+{H}_{pump}-{H}_{0}$$

26 $$M=\sqrt{{Y}^{2}-4WZ}$$

The submergence buildup curve can be drawn through the mathematical relationship between submergence and time. After the obtained submergence buildup curve is evenly divided into N intervals, the slope of each interval can be determined from coordinates of two adjacent points are recorded ((t_3_, h_3_) and (t_4_, h_4_) in Fig. 5). PU starts working when the absolute value of the slope is small than a certain value (ε_2_), i.e.; $|\frac{{h}_{3}-{h}_{4}}{{t}_{3}-{t}_{4}}|\le {\varepsilon }_{2}$; and this moment is recorded as T_2_ as shown in Fig. 4. The calculation method of ε_2_ is the same as the ε_1_.

**Figure 6:**
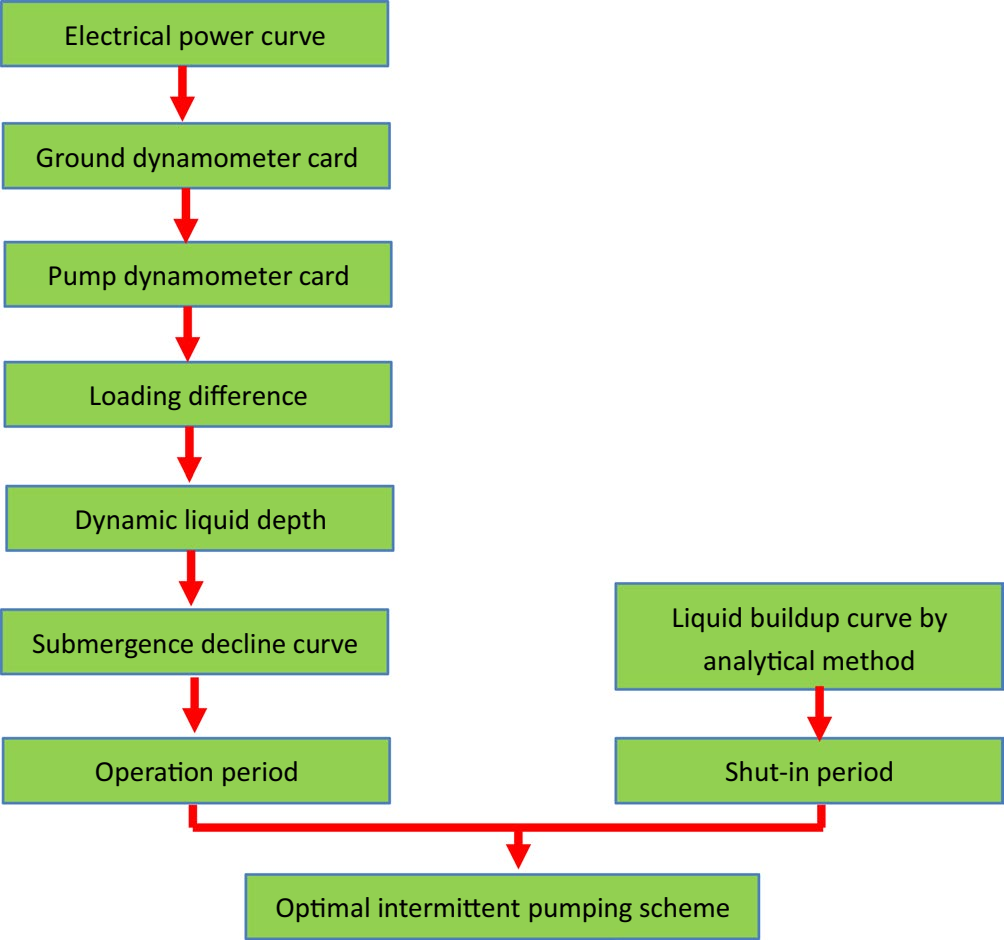
Workflow diagram to determine the optimal intermittent pumping scheme.

## Results: Case Study for Field Application

According to the method mentioned above, a flow chart (Fig. 6) and software have been developed. Inputting the working conditions of a PU, the software can optimize the intermittent pumping scheme in real-time.

A well from Jilin oilfield was chosen as an example. The parameters are shown in Table 1 and Table 2.

**Table 1:** Production data.

Parameters	Value
Tubing Pressure(MPa)	0.4
Casing Pressure(MPa)	0.2
Relative Water Density(dimensionless)	1.0
Relative Gas Density(dimensionless)	0.64
Relative Oil Density(dimensionless)	0.8
Pump Depth(m)	1573
The Diameter of Pump(mm)	38
Output before Intermittent (t/d)	2
Water Cut	0.4
Stroke (m)	2
Stroke Speed (min^−1^)	2.9
Gas-Oil Ratio(dimensionless)	30
Bubble Pressure (MPa)	6.5
Depth of Layer (m)	3000
The Temperature in the Bottom of Well (K)	353

**Table 2:** Parameters of suction rod and motor.

Parameters	Value
Inner Diameter of Tubing (mm)	62
Outer Diameter of Tubing (mm)	73
Inner Diameter of Casing (mm)	127.3
Outer Diameter of Casing (mm)	139.7
Level of Rod(Grade)	3
Length of First Rod (m)	732
The Diameter of First Rod (mm)	22
Length of Second Rod (m)	675
The Diameter of Second Rod (mm)	19
Length of Third Rod (m)	267
The Diameter of Third Rod (mm)	38
Type of Pumping Unit	CYJ8-3-37HF
Motor Power (kW)	16
Motor Transmission Ratio(dimensionless)	30.48
Motor Speed (r/min)	860
Fluid Rates through Valve (m/s)	0.15

After inputting the above parameters to the software, and intermittent pumping scheme has been optimized. The result turns out that the operation period and the shut-in period is 17 and 10 hours, respectively. The current production rate is 1.9 tons per day and the profit is 31,000 RMB. Comparing to the former pumping scheme with the operation period of 22 hours and a shut-in period of 8 hours, the production rate is slightly decreased by 5%, but the profit is increased by 25%. This is attributed to the decreased energy expense when the optimized intermittent pumping scheme is applied. In general, this method has a promising future for maximizing the profits from old oilfields with fluid-shortage wells.

## Conclusion

An intermittent pumping scheme for fluid shortage wells has been developed in this study. The variation law of annulus dynamic fluid depth has been studied to design the optimal intermittent pumping scheme.

1. By studying the relationship between electrical power, torque, and loading, the surface dynamometer card can be interpreted from electrical power curve; this is used to calculate the dynamic fluid level.
2. During the operation and shut-in periods, both decreasing and rising rates of submergence are fast in the beginning and approaching a plateau in the end. The optimal operation and shut-in time are obtained from the inflection points of their variation curves.
3. The determination of the shut-in period can be solved by the mathematical relationship between inflow performance and submergence buildup law.
